# Five novel *NF1* gene pathogenic variants in 10 different Chinese families with neurofibromatosis type 1

**DOI:** 10.1002/mgg3.904

**Published:** 2019-07-25

**Authors:** Linlin Chen, Feng Xue, Jia Xu, Jinwei He, Wenzhen Fu, Zhenlin Zhang, Qinglin Kang

**Affiliations:** ^1^ Department of Orthopedic Surgery Shanghai Jiao Tong University Affiliated Sixth People's Hospital Shanghai China; ^2^ Medical College of Soochow University Suzhou China; ^3^ Metabolic Bone Disease and Genetic Research Unit, Department of Osteoporosis and Bone Diseases Shanghai Jiao Tong University Affiliated Sixth People's Hospital Shanghai China

**Keywords:** CPT, Neurofibromatosis type 1, *NF1* gene, Pathogenic variant

## Abstract

**Background:**

Neurofibromatosis type 1 (NF1) is an autosomal dominant disorder with equal sex incidence that is characterized by neurofibromas, café‐au‐lait macules, axillary freckling, optic pathway tumor, distinctive osseous lesion, and iris Lisch nodules. Inactivating variants in the *NF1* gene have been identified to be correlated with NF1. This tumor suppressor gene is located at 17q11.2.

**Methods:**

Ten affected NF1 probands and their available relatives from 10 unrelated Chinese families with neurofibromatosis type 1 were clinically studied. All of these probands mainly complained of osseous lesions. PCR was used to analyze and sequence the variants. We collected both laboratory and radiological information.

**Results:**

We detected five novel pathogenic variants including two de novo variants in these 10 families: one missense variant, p.Cys709Arg(c.2125T>C), in exon 18 and four frameshift variants: p.Leu1459Profs*2(c.4436dupT) in exon 34; p.Lys99Argfs*4(c.296delA) in exon 4; p.Leu762Cysfs*2(c.2283delA) in exon 19; and p.Leu1522Ilefs*53(c.4562_4563dupAT) in exon 34.

**Conclusion:**

Novel pathogenic variants in the *NF1* gene in these families correlated with the phenotype and genotype and explained the clinical manifestations of these patients. The results help us to understand the genetic basis of patients with neurofibromatosis type 1 in China. Our study expands the pathogenic variant spectrum of the *NF1* gene and may be helpful in genetic counseling and prenatal genetic diagnosis.

## 1 INTRODUCTION

Neurofibromatosis type 1 (NF1; MIM 162200) is an inherited autosomal dominant disorder that is accompanied by abnormalities in multiple tissues derived from the neural crest with a prevalence of approximately 1 per 3,000 (Friedman, 1999; Wallace et al., 1990). The National Institutes of Health (NIH) consensus conference established diagnostic criteria for NF1. It is characterized by a plethora of clinical features, including neurofibromas, axillary and inguinal freckling, diffuse café‐au‐lait macules, optic pathway tumors, distinctive osseous lesions, and iris Lisch nodules (Ferner et al., 2007; Rasmussen & Friedman, 2000). Patients with *NF1* gene pathogenic variants also exhibit other phenotypes, including neurocognitive deficits, and cardiovascular abnormalities (Williams et al., 2009). Patients with NF1 have an 8%–13% risk of developing malignant peripheral nerve sheath tumors (MPNSTs) in their lifetime (Evans et al., 2002).

NF1 generally occurs due to inactivating variants in the *NF1* gene which is a tumor suppressor gene located at 17q11.2 (Upadhyaya et al., 2012). Neurofibromin is encoded by the *NF1* gene, which contains 58 exons distributed over 350 Kb of the genomic DNA (Peltonen, Kallionpaa, & Peltonen, 2017; Prasad, Chandra, Sudarsan, Kumar, & Sarma, 2018; Trovo‐Marqui & Tajara, 2006). Neurofibromin has a Ras‐GAP (GTPase‐Activating‐Protein) domain and is mainly expressed in neurons, Schwann cells, and oligodendrocytes in the nervous system (Cichowski & Jacks, 2001; Daston et al., 1992). It is also expressed in skeletal cells and tissues, including chondrocytes, osteoblasts, osteocytes, and osteoclasts (Kuorilehto, Nissinen, Koivunen, Benson, & Peltonen, 2004). To date, more than 2,900 *NF1* gene pathogenic variants without obvious mutational hot spots have been reported in the Human Gene Mutation Database (http://www.hgmd.org). Approximately 50% of all NF1 cases are sporadic and present as de novo variants (Korf, 2013). This study is aimed at analyzing both the laboratory and radiographic information in these Chinese patients and understanding the *NF1* gene pathogenic variants that occurred in these Chinese families.

## 2 MATERIALS AND METHODS

### 2.1 Ethical Compliance

The Ethics Committee of the Shanghai Jiao Tong University Affiliated Sixth People's Hospital approved this project. Before participating in the study, patients or their legal guardians have written informed consent.

### 2.2 Materials

**Figure 1:**
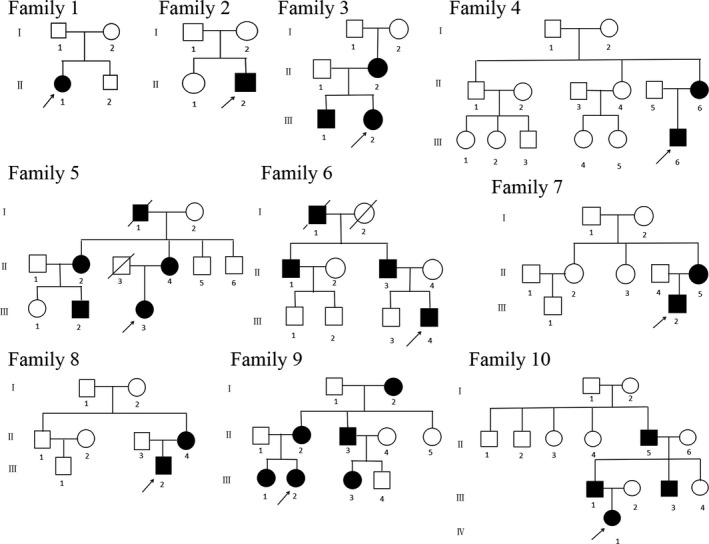
Pedigrees of families with Neurofibromatosis type 1. The black symbols represent the affected individuals, and the open symbols represent the unaffected individuals. The circles and squares indicate females and males, respectively. The arrows identify the probands in the families

We obtained clinical data and peripheral blood samples. Ten families with NF1 were investigated in our study, and all were of the Chinese Han ethnicity but from different regions. Ten probands and 18 relatives of these families were diagnosed with NF1 based on the diagnostic criteria established by the National Institutes of Health (NIH). All these cases are previously unreported. Pedigrees of the 10 families with NF1 are shown in Figure 1. The clinical manifestations and laboratory examinations are shown in Table 1.

**Table 1:** Clinical characteristics of NF1 patients studied

Family No.	Patient No.	Age (year)	Gender	CALMs	Neurofibromas	Freckling	Optic glioma	Lisch nodules	Osseous lesions	Intellectual disability
1	II−1	15	F	+	−	−	−	−	CPT, scoliosis	−
2	II−2	23	M	+	+	+	−	−	CPT, decreased BMD	−
3	III−2	7	F	+	−	+	−	−	CPT	−
3	II−2	43	F	+	−	+	NA	NA	−	−
3	III−1	20	M	+	−	+	NA	NA	−	−
4	III−6	19	M	+	−	+	−	−	CPT, scoliosis	−
4	II−6	41	F	+	−	+	NA	NA	−	−
5	III−3	18	F	+	+	+	−	−	CPT	−
5	II−4	42	F	+	+	+	NA	NA	−	−
6	III−4	14	M	+	−	+	−	−	Unequal leg length	−
6	II−3	50	M	+	+	+	NA	NA	−	−
7	III−2	3	F	+	−	+	−	−	Unequal leg length	−
7	II−5	24	F	+	−	+	NA	NA	−	−
8	III−2	12	M	+	−	−	−	−	Scoliosis	−
8	II−4	40	F	+	−	+	NA	NA	−	−
9	III−2	12	F	+	−	+	−	−	CPT, decreased BMD	−
9	II−2	45	F	+	+	+	NA	NA	−	−
9	III−1	24	F	+	+	+	NA	NA	−	−
10	IV−1	3	F	+	−	+	−	−	CPT	−
10	III−1	38	M	+	+	+	NA	NA	−	−
10	II−5	67	M	+	+	+	NA	NA	−	−

Abbreviations: BMD, bone mineral density; CALMs café‐au‐lait, macules; CPT, congenital pseudarthrosis of the tibia; F, female, M male, NA, not available.

In family 1, the 15‐year‐old proband (II‐1) was a girl from the Anhui province. She was born to a healthy nonconsanguineous couple. The girl had exhibited tibial bowing since she was 1 month old. When she was 18 months old, her left tibia fractured in the absence of any obvious predisposing factors. At the time of presentation to us, she had congenital pseudarthrosis of the tibia (CPT) (Figur2a), café‐au‐lait macules, and mild scoliosis. Her vision and hearing were normal. The bone mineral density of both the spine and total hips were normal. She was 151cm in height at the time of the study. In the rest of the family members, no similar clinical abnormalities were observed.

**Figure 2:**
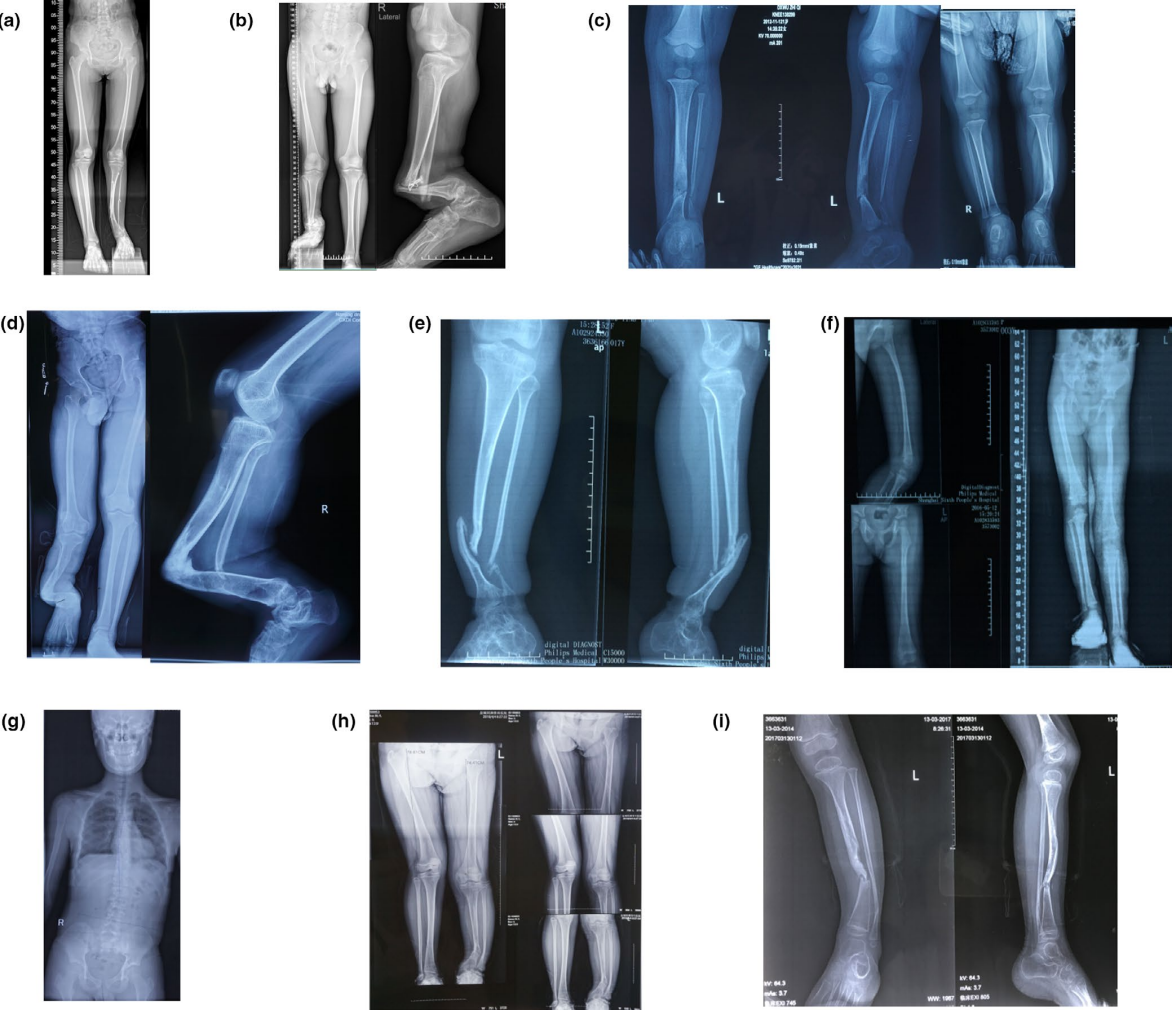
The radiological evidence of NF1 in our patients. (a) II‐1 of family 1: the radiograph demonstrates her congenital pseudarthrosis of the left tibia. (b) II‐2 of family 2: the radiographs demonstrate his congenital pseudarthrosis of the right tibia. (c) III‐2 of family 3: the radiographs demonstrate her congenital pseudarthrosis of the left tibia. (d) III‐6 of family 4: the radiographs demonstrate his congenital pseudarthrosis of the right tibia. (e) III‐3 of family 5: the radiographs demonstrate her congenital pseudarthrosis of the left tibia. (f) III‐2 of family 7: the radiographs demonstrate his unequal leg length deformity. (g) II‐2 of family 8: the radiograph demonstrates his scoliosis. (h) III‐2 of family 9: the radiograph demonstrates her tibial bowing deformity. (i) IV‐1 of family 3: the radiographs demonstrate her congenital pseudarthrosis of the left tibia. All of the images are published with permission from the affected individuals

In family 2, the 23‐year‐old proband (II‐2) was a man from the Anhui province who was born of a nonconsanguineous marriage. Since he was 2 years old, he had experienced several times fractures in the right tibia and fibula. He also presented with CPT (Figur2b), axillary and inguinal freckling, and café‐au‐lait macules. His right eye exhibited blepharoptosis but without optic gliomas or Lisch nodules. He also showed a decreased BMD (bone mineral density). He was 165 cm in height at the time of the study. No family history of these symptoms was identified.

In family 3, the 6‐year‐old proband (III‐2) referred to our hospital was a girl from the Chongqing province and she was born to a nonconsanguineous couple. She had exhibited tibial bowing (Figur2c) since birth. Her birth weight and length were within normal limits. She was 95cm in height at the time of the study. She also presented with axillary and inguinal freckling, and café‐au‐lait macules. Both her mother (II‐2) and older brother (III‐1) had similar symptoms but without tibial bowing.

In family 4, the 19‐year‐old proband (III‐6) was a man from the Anhui province and he was born to a nonconsanguineous couple. The man had exhibited CPT (Figur2d) since he was born. At the time of presentation to us, he also had café‐au‐lait macules, freckling, and scoliosis. Since he was 2‐year‐old, he had experienced several times fractures in the right tibia and fibula due to minor violence. He was 170cm in height at the time of the study. His mother (II‐6) also presented with café‐au‐lait macules and freckling. However, her mother did not have tibial bowing and never experienced any fracture.

In family 5, the 15‐year‐old proband (III‐3) who presented to our hospital was a girl from the Zhejiang province with a chief complaints of CPT (Figur2e). She had dozens of neurofibromas, café‐au‐lait macules, and axillary and inguinal freckling. At the age of 6 months, she had experienced her first fracture in the left tibia and fibula for no apparent reason. To date, she has had 12 times fractures in the left tibia and fibula. She was 150cm in height at the time of the study. No family history of consanguineous marriage or bone fragility was identified. Her mother (II‐4) also presented with café‐au‐lait macules and freckling. However, her mother has never experienced any fractures.

In family 6, the 14‐year‐old proband (III‐4) referred to our hospital was a boy from the Jiangxi province and his parents were nonconsanguineous. At the age of 8 years old, he underwent an operation to remove the giant neurofibromas near his left hip. He had dozens of café‐au‐lait macules and some axillary and inguinal freckling. He had never suffered from any fractures. He was 148cm in height at the time of the study. His grandfather (I‐1), father (II‐3), and uncle (II‐1) had similar symptoms but without osseous lesions. Unfortunately, his X‐ray results before the operation were not available.

In family 7, the 3‐year‐old proband (III‐2) from the Hebei province was the only child of a nonconsanguineous couple. This girl had exhibited café‐au‐lait macules and freckling since she was 2 months old. Beginning at 2 years of age, her parents noticed progressive limb length discrepancy of her legs (Figur2f). She was 90cm in height at the time of the study. Her mother (II‐5) had similar symptoms but without limb length discrepancy.

In family 8, the 12‐year‐old proband (III‐2) was a boy from the Jilin province. The boy was the only child of a nonconsanguineous couple. He was referred to our hospital with a chief complaints of scoliosis (Figur2. G) and café‐au‐lait macules across his back. He was 153cm in height at the time of the study. His mother (II‐4) had similar symptoms but without scoliosis. His father and his grandparents showed no signs of NF1.

In family 9, the 12‐year‐old proband (III‐2) was a girl from the Jiangsu province and she was born to a nonconsanguineous couple. The girl had exhibited tibial bowing (Figur2h) since she was 5 years old. At the time of presentation to us, she also had café‐au‐lait macules and freckling. She was 154cm in height at the time of the study. Her grandmother (I‐2), mother (II‐2), and older sister (III‐1) had similar symptoms but without tibial bowing.

In family 10, the 3‐year‐old proband (IV‐1) from the Zhejiang province was the only child of a nonconsanguineous couple. The girl had exhibited tibial bowing (Figur2i) since she was 1 year old. At the age of 2 years, she had experienced a fracture in the left tibia and fibula. She presented to our hospital with café‐au‐lait macules across her body. Her left eye exhibited blepharoptosis and proptosis but without optic glioma or Lisch nodules. She was 91cm in height at the time of the study. Her grandfather (III‐5) and father (III‐1) showed café‐au‐lait macules, neurofibromas, freckling. Her uncle (III‐3) had only café‐au‐lait macules.

### 2.3 Methods

By using a conventional extraction method, we isolated DNA from peripheral leukocytes which were collected from patients in ethylenediaminetetraacetic acid (EDTA) tubes. NF1 gene variants were screened among the 10 probands, other members of the 10 families, and 200 unrelated, healthy controls (males: 100, females: 100). We obtained the DNA sequence of the NF1 gene from the online database (GenBank accession NO. NC_000017.11). Using Primer‐3 software (http://bioinfo.ut.ee/primer3-0.4.0/)was used to design the primers (54 pairs)(Zhu et al., 2016). Then we amplified all 58 exons and their exon–intron boundaries in the NF1 gene by polymerase chain reaction (PCR). For fragments NF1‐1F2/1R2, 1F3/1R3, and 1F4/1R4, the reaction mixture (20µl) included 1x GC buffer I (TAKARA), 2.5 mM Mg2+, 0.2 mM dNTP, 0.2 µM of each primer, 1 U HotStarTaq polymerase (TAKARA) and 1 µl template DNA. The cycling program was 95ºC for 2 min; 42cycles x (96ºC for 10 s, 70ºC for 2 min); and 4ºC indefinitely. For the other 51 fragments, the reaction mixture (20µl) included 1x GC buffer I (TAKARA), 2.5 mM Mg2+, 0.2 mM dNTP, 0.2 µM of each primer, 1 U HotStarTaq polymerase (TAKARA) and 1 µl template DNA. The cycling program was 95ºC for 2 min; 11 cycles x (94ºC for 20s, 62ºC–0.5ºC/cycle for 40 s, 72ºC for 1 min); 28 cycles x (94ºC for 20s, 56ºC for 30s, 72ºC for 1 min); 72ºC for 2 min; and 4ºC indefinitely. A total of 0.5 U SAP and 4 U Exo I were added into 8 µl PCR products. The mixture was incubated at 37ºC for 60 min, followed by incubation at 75ºC for 15 min. The reaction mixture included 3 µl BigDye3.1 mix, 2 µl sequencing primer (1 µM), and 1–2 µl purified PCR product. The cycling program was 96ºC for 1 min; 28 cycles x (96ºC for 10 s, 50ºC for 5 s, 60ºC for 4 min); and 4ºC indefinitely. Direct sequencing was performed using a BigDye Terminator Cycle Sequencing Ready Reaction Kit, version 3.1 (Applied Biosystems), and the sequences were analyzed with an ABI Prism 3,130 automated sequencer. Single‐nucleotide polymorphisms (SNPs) were identified using Polyphred (http://droog.mbt.washington.edu/poly_get.html), and novel pathogenic variants were identified using Mutalyzer 2.0 (http://mutalyzer.nl/ check) and HGMD (http://www.hgmd.cf.ac.uk/). Any changes that occurred in the amino acids with NCBI frequency information lower than CHBS0.01 in the 1000G database signified as a pathogenic variant site.

## 3 RESULTS

In our study, we report the results of variant screening for the NF1 gene in 10 families. Genetic findings and analyses are shown in Table 2. All of these variants were heterozygous, and five were novel variants.

**Table 2:** NF1 variants identified in this study

Family number	Variant position	Nucleotide change	Amino acid change	Variant type	References	Inheritance	Conservative analysis (UniProt)	Bioinformatics prediction (mutation taster)	Bioinformatics prediction (PolyPhen‐2)
1	Exon34	c.4436dupT	p.Leu1480Profs*2	Insertion	Novel	Sporadic	Fully conserved	Disease causing	NA
2	Exon4	c.296delA	p.Lys99Argfs*4	Deletion	Novel	Sporadic	Fully conserved	Disease causing	NA
3	Exon46	c.6855C>A	p.Tyr2285*	Nonsense	Reported	Familial	Fully conserved	Disease causing	NA
4	Exon19	c.2283delA	p.Leu762Cysfs*2	Deletion	Novel	Familial	Fully conserved	Disease causing	NA
5	Exon18	c.2125T>C	p.Cys709Arg	Missense	Novel	Familial	Fully conserved	Disease causing	Probably damaging (0.999)
6	Exon34	c.4562_4563dupAT	p.Leu1522Ilefs*53	Insertion	Novel	Familial	Fully conserved	Disease causing	NA
7	Intron32	c.4332+1G>T	Putative aberrant splicing	Splicing	Reported	Familial	NA	NA	NA
8	Exon29	c.3916C>T	p.Arg1306*	Nonsense	Reported	Familial	Fully conserved	Disease causing	NA
9	Exon37	c.4936A>C	p.Thr1646Pro	Missense	Reported	Familial	Fully conserved	Disease causing	Probably damaging (1.000)
10	Exon4	c.574C>T	p.Arg192*	Nonsense	Reported	Familial	Fully conserved	Disease causing	NA

Note

Reported Indicates that the mutation has been reported in the Human Gene Mutation Database (HGMD). All variants are located according to NM_001042492.2.

Abbreviations: NA, not applicable; PolyPhen‐2, Polymorphism Phenotyping version 2.

In family 1, we identified a frameshift variant in the proband (II‐2). It was a c.4436dupT of exon 34. This frameshift variant inserted a thymidine that changed the codon TCC to TTC and then resulted in the subsequent change of a leucine codon to a proline codon at p.1459 and truncation at p.1461 (Figur3a).

**Figure 3:**
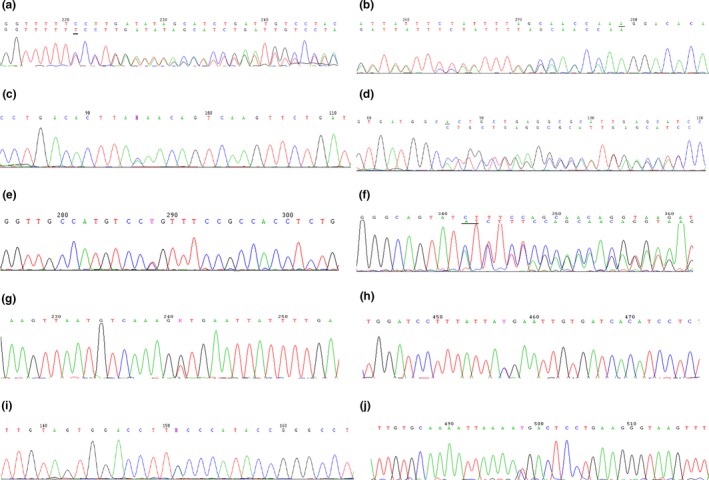
Mutational analysis. (a) A frameshift variant, c.4436dupT in exon 34 of the NF1 gene, was found in proband II‐1 of family 1. (b) A frameshift variant, c.296delA in exon 4 of the NF1 gene, was found in proband II‐2 of family 2. (c) A nonsense variant, c.6855C>A in exon 46 of the NF1 gene, was found in proband III‐2, III‐1 and their mother II‐2 of family 3. (d) A frameshift variant, c.2283delA in exon 19 of the NF1 gene, was found in proband III‐6 and her mother II‐6 of family 4. (e) A missense variant, c.2125T>C in exon 18 of the NF1 gene, was found in proband III‐3, III‐2, II‐2, II‐4, and I‐1 of family 5. (f) A frameshift variant, c.4562_4563dupAT in exon 34 of the NF1 gene, was found in proband III‐4, II‐1, II‐3, and I‐1 of family 6. (g) A splicing variant, c.4332+1G>T in Intron 32 of the NF1 gene, was found in proband III‐2 and his mother II‐5 of family 7. (h) A nonsense variant, c.3916C>T in exon 29 of the NF1 gene, was found in proband III‐2 and his mother II‐4 of family 8. (i) A missense variant, c.4936A>C in exon 37 of the NF1 gene, was found in proband III‐2, III‐1, III‐3, II‐2, II‐3, and I‐2 of family 9. (j) A nonsense variant, c.574C>T in exon 5 of the NF1 gene, was found in proband IV‐1, III‐1, III‐3, and II‐5 of family 10

In family 2, we found a frameshift variant in the proband (II‐2). It was a c.296delA of exon 4. This frameshift variant deleted an adenine that changed the codon AAG to AGG and then resulted in the subsequent change of a lysine codon to an arginine codon at p.99 and truncation at p.103 (Figur3b).

In family 3, we found a nonsense variant in the proband (III‐2), her mother (II‐2) and older brother (III‐1). This nonsense variant of exon 46 was a C‐to‐A transition at c.6792. It changed codon TAC to TAA and transformed a tyrosine codon to a stop codon, resulting in truncation at p.2265 (Figur3c).

In family 4, we found a frameshift variant in the proband (III‐6)and his mother (II‐6). It was a c.2283delA of exon 19. This frameshift variant deleted an adenine that changed the codon CTG to TGC and then resulted in the subsequent change of a leucine codon to a cystine codon at p.762 and truncation at p.764 (Figur3d).

In family 5, we detected a missense variant in the proband (III‐3)and her mother (II‐4). This missense variant of exon 18 was a T‐to‐C transition at c.2125 and then resulted in a cystine (TGT)‐to‐arginine (CGT) substitution at p.709 (Figur3e).

In family 6, we detected a frameshift variant in the proband (III‐4). It was a c.4562_4563dupAT of exon 34. This frameshift variant inserted a thymidine and a thymine which changed the codon CTT to ATC and then resulted in the subsequent change of a leucine codon to an isoleucine codon at p. 1501 and truncation at p.1554 (Figur3f).

In family 7, we found a splicing variant in the proband (III‐2)and her mother (II‐5). This splicing variant of intron 32 was a heterozygous c.4332 + 1G>T that resulted in putative aberrant splicing (Figur3g).

In family 8, we found a nonsense variant in the proband (III‐2) and his mother (II‐4). This nonsense variant of exon 29 was a C‐to‐T transition at c.3916. It changed codon CGA to TGA and then transformed an arginine codon to a stop codon, resulting in truncation at p.1307 (Figur3H).

In family 9, we identified a recurrent missense variant in the proband (III‐2). This missense variant of exon 37 was a heterozygous A‐to‐C transition at c.4873, resulting in a threonine (ACC+++)‐to‐proline (CCC) substitution at p. 1625 (Figur3I).

In family 10, we detected a nonsense variant in the proband (IV‐1)and her father (III‐1). This nonsense variant of exon 5 was a C‐to‐T transition at c.574. It changed codon CGA to TGA and then transformed an arginine codon to a stop codon, resulting in truncation at p.1307 (Figur3J).

None of these above *NF1* variants were found in DNA samples from unaffected family members or 200 unrelated healthy volunteers.

## 4 DISCUSSION

Neurofibromatosis type 1 (NF1) is characterized by obvious age‐related penetrance and wide clinical variability, but almost all adults with NF1 present with cutaneous and/or subcutaneous neurofibromas (Koczkowska et al., 2019; Uusitalo et al., 2015). Multiple osseous lesions including CPT, tibial dysplasia, scoliosis, and decreased bone mineral density (BMD) are related to neurofibromatosis type 1 (Stevenson et al., 2011). Fifty percent of NF1 patients suffer from osseous lesions, and these can be debilitating in some cases (Crawford & Bagamery, 1986).

In our current study, 10 different unrelated Chinese families were enrolled, and probands in these families were clinically diagnosed with NF1 according to guidelines established by the NIH. We found 10 different *NF1* gene pathogenic variants (five of them were novel pathogenic variants) in these unrelated families. All of these probands mainly complained of osseous lesions. Seven probands exhibited CPT, three of them exhibited scoliosis, two of them exhibited unequal leg length, two of them exhibited unequal leg length, and two of them exhibited decreased bone mineral density (BMD). According to some studies, the incidence of pseudarthrosis in NF1 patients presenting with bowing of the long bones is higher, and NF1 loss of heterozygosity (LOH) detection has been tested in these fracture sites of pseudarthrosis tissue (Stevenson et al., 2011). The development of tibial pseudarthrosis is postulated to be related to double inactivation of NF1, but the mechanism of the “second hit” mutation remains unclear (Sant et al., 2015).

The nonsense variants p.Tyr2285* in exon 46, p.Arg1306* in exon 29, and p.Arg192* in exon 4, lacking exons 46 to 58, exons 29 to 58, and exons 4 to 58, respectively may result in the translation of truncated proteins. We detected two missense variants: p.Cys709Arg in exon 18 and p.Thr1646Pro in exon 37. Two deletion variants were identified in our study: p.Lys99Argfs*4 in exon 4 and p.Leu762Cysfs*2 in exon 19. Two insertions were detected: p.Leu1480Profs*2 in exon 34 and p.Leu1522Ilefs*53 in exon 34. We identified one splice site variant at introns 32 that was predicted to cause putative aberrant splicing. This splicing would result in truncated proteins due to the reading frame shift that caused by the pathogenic variant.

The NF1 gene variants detected in the two sporadic patients (in families 1 and 2) whose parents are healthy are most likely de novo variants. Instead of inheriting them from the parents, these variants could occur in the germ cell or its mosaicism in the fertilized egg itself or in the gonads of one of the parents.

Although, there is no obvious genotype or phenotype correlation was demonstrated in our study, we detected five novel pathogenic variants and described different clinical features. The identification of these variants provides a basis for further study of NF1 and for genetic counseling and prenatal diagnosis. Functional studies of NF1 gene variant need to be performed to expound on how genotype correlate to phenotype.

## CONFLICTS OF INTEREST

None.

5

## Data Availability

Data are available to share.
